# Consensus in rooted dynamic networks with short-lived stability

**DOI:** 10.1007/s00446-019-00348-0

**Published:** 2019-02-13

**Authors:** Kyrill Winkler, Manfred Schwarz, Ulrich Schmid

**Affiliations:** TU Wien, Vienna, Austria

**Keywords:** Dynamic networks, Consensus, Message adversary, Eventual stability, Short stability periods, Rooted directed graphs

## Abstract

We consider the problem of solving consensus using deterministic algorithms in a synchronous dynamic network with unreliable, directional point-to-point links, which are under the control of a message adversary. In contrast to the large body of existing work that focuses on message adversaries that pick the communication graphs from a predefined set of candidate graphs arbitrarily, we consider message adversaries that also allow to express eventual properties, like stable periods that occur only eventually. Such message adversaries can model systems that exhibit erratic boot-up phases or recover after repeatedly occurring, massive transient faults. We precisely determine how much eventual stability is necessary and sufficient, and provide an optimal consensus algorithm. Unlike in the case of longer stability periods, where standard algorithms can be adapted for solving consensus, different algorithmic techniques are needed in the case of short-lived stability.

## Introduction

We consider deterministic consensus algorithms in synchronous dynamic networks, where a potentially unknown number of processes that never fail^1^ communicate via unacknowledged messages over unreliable point-to-point links. Consensus, which is a pivotal service in truly distributed applications, is the problem of computing a common decision value based on local input values of all the processes. An execution of a consensus algorithm in our system proceeds in a sequence of lock-step synchronous^2^ rounds, where message loss is modelled using an omniscient message adversary that determines the directed *communication graph*${\mathcal {G}}^r$ for each round *r*. A directed edge $(p \rightarrow q)$ present in ${\mathcal {G}}^r$ means that the message sent by *p* in round *r* is successfully received by *q* in the same round.

In most existing work in this area, e.g. [1, 11, 22, 23], the message adversary may choose each ${\mathcal {G}}^r$ from the *same* set of admissible graphs arbitrarily in each round. For instance, the classic result from Santoro and Widmayer [22] states that consensus is impossible if the adversary may suppress up to $n-1$ messages in every round. More recently, [11] introduced an equivalence relation on the set of admissible communication graphs such that consensus is solvable if and only if for each equivalence class there is a common *source* (a node that has a directed path to every other node) in every graph. These (and similar) approaches characterize the solvability of consensus by means of properties of the *set* of admissible graphs.

We also explore the solvability/impossibility border of consensus, albeit under message adversaries that support *eventual stabilization* [3, 4, 24]: Here, the set of admissible choices for ${\mathcal {G}}^r$ may change with evolving round numbers *r*. Rather than constraining the set of admissible graphs, we hence constrain admissible graph *sequences*. As it turns out, consensus can be solved for graph sequences where the *set* of graphs occurring in the sequence would render consensus impossible under a message adversary where only the set of admissible graphs for each round is restricted [11, 22].

Apart from being theoretically interesting, considering eventually stabilizing dynamic networks is also useful from a practical perspective: algorithms that work correctly under eventually stabilizing message adversaries are particularly suitable for systems that suffer from uncoordinated boot-up sequences or systems that must recover from massive transient faults: Network connectivity can be expected to improve over time here, e.g., due to improving clock synchronization quality. Since it is usually difficult to determine the time when such a system has reached normal operation mode, algorithms that terminate only after a reasonably stable period has been reached are obviously advantageous. Algorithms that work correctly under short-lived stable periods are particularly interesting, since they have higher coverage and terminate earlier in systems where longer stable periods occur only rarely or even not at all. Note that the occurrence of short-lived stability periods were confirmed experimentally in the case of a prototype wireless sensor network [20].

Last but not least, stabilizing algorithms require less reliable and, in our case, not inherently bidirectional communication underneath, hence work with cheaper and/or more energy-efficient network communication interfaces. After all, guaranteeing reliable bidirectional communication links typically incurs significant costs and/or delays and might even be impossible in adverse environments. We hence conjecture that our findings may turn out useful for applications such as mobile ad-hoc networks [15] with heavy interference or disaster-relief applications [18].

In view of such applications, our core assumption of a synchronous system may appear somewhat unreasonable. However, it is not thanks to modern communication technology [25]: As synchronized clocks are typically required for basic communication in wireless systems anyway, e.g., for transmission scheduling and sender/receiver synchronization, global synchrony is reasonably easy to achieve: It can be integrated directly at low system levels as in 802.11 MAC+PHY [14], provided by GPS receivers, or implemented by means of network time synchronization protocols like IEEE 1588 or FTSP [19].

*Main contributions and paper organization* In this paper, we thoroughly answer the question of the minimal stability required for solving consensus under eventual stabilizing message adversaries. After the introduction of our system model and our message adversaries in Sects. 2 and 3, respectively, we establish the following results:

1. We provide a novel algorithm in Sect. 5, along with its correctness proof, which solves consensus for a message adversary that generates graph sequences consisting of graphs that (i) are rooted, i.e., have exactly one root component (a strongly connected component without incoming edges from outside of the component), and (ii) contain a subsequence of $x=D+1$ consecutive graphs whose root component is formed by the same set of nodes (“stable root component”). Herein, the system parameter $D \leqslant n-1$ is the dynamic network depth, i.e., the number of rounds required for all nodes in a stable root component to reach all nodes in the system. Thanks to (i), our algorithm is always safe in the sense that agreement is never violated; (ii) is only needed to ensure termination. Compared to all existing algorithms for message adversaries like [3, 4, 24], where the processes more or less wait for the stability window to occur, our algorithm employs new algorithmic techniques once a candidate stability window has been found: We show that by “waiting for contradictory evidence”, the repeated information propagation from one process to the entire system is enough for everyone to reliably determine if the candidate window was just spurious and should be discarded, or if it has to be taken seriously, because someone is convinced that it was the guaranteed stability window.
2. In previous work [3, 5], it has been shown that $x=D-1$ is a lower bound for the stability interval for all consensus algorithms working under message adversaries that guarantee a stable root component to occur eventually, and that (a bound on) *D* must be known a priori.^3^ In Sect. 4 of this paper, we improve the lower bound to $x=D$, which reveals that the previous bound was not tight and that our algorithm is optimal. This result also shows that the mere propagation of an input value to every process during the stability window does not suffice to solve consensus in this setting.

Some conclusions and directions of future work in Sect. 6 complete the paper.

### Related work

Research on consensus in synchronous message passing systems subject to link failures dates back at least to the seminal paper [22] by Santoro and Widmayer; generalizations have been provided in [6, 9–11, 23]. In all these papers, consensus, resp. variants thereof, are solved in systems where, in each round, a digraph is picked from a set of possible communication graphs. The term message adversary was coined by Afek and Gafni in [1] for this abstraction.

A different approach for modeling dynamic networks has been proposed in [16]: *T*-interval connectivity guarantees a common subgraph in the communication graphs of every *T* consecutive rounds. [17] studies agreement problems in this setting. Note that solving consensus is relatively easy here, since the model assumes bidirectional and always connected communication graphs. In particular, 1-interval-connectivity, the weakest form of *T*-interval connectivity, corresponds to all nodes constituting a perpetually constant set of source nodes.

In both lines of research, there is no notion of eventually stabilizing behavior of dynamic networks. To the best of our knowledge, the first instance of a message adversary that guarantees eventual stable root components has been considered in [3]: It assumed communication graphs with a non-empty set of sources and long-living periods of stability $x=4D+1$. [4] studies consensus under a message adversary with comparably long-lived stability, which gracefully degrades to general *k*-set agreement in case of unfavorable conditions. However, this message adversary must also guarantee a certain influence relation between subsequently existing partitions. [24] established a characterization of uniform consensus solvability/impossibility for longer stability periods. In particular, it provides a consensus algorithm that works for stability periods of at least $2D+1$ but does not require graph sequences where all graphs are rooted. Note that the experimental evaluation of a wireless sensor network described in [20] reveals that this assumption holds true, for a properly chosen value of *D* (in particular, $D=4$), with a coverage close to 100% both in an indoor and outdoor environment. Whereas one cannot obviously generalize from a single, non-systematic experimental evaluation, these findings nevertheless suggest that the basic assumption of an eventually vertex-stable root component is not unreasonable in practice.

Finally, [21] used message adversaries that allow a notion of “eventually forever” to establish a relation to failure detectors. Although we do not consider this “extremal” case in this paper, which solely addresses short-lived stability, we note that interesting insights can be drawn from this relation [5].

Finally, we note that our whole approach of designing algorithms tailored to a particular message adversary is in stark contrast to the approach advocated in [21], which shows, among other insightful results, that the message adversary ${\text {SOURCE}}+{\text {QUORUM}}$ allows to simulate an asynchronous message passing system with process crashes augmented by the failure detector $({\varSigma },{\varOmega })$. A failure detector is an oracle that can be queried by all processes in order to gain some information on failures (traditionally process crashes) that could otherwise not be obtained. They have been introduced as an abstraction to capture precisely what additional power is necessary to solve consensus in a system where consensus is ordinarily impossible (see [8] for a more thorough introduction). Since $({\varSigma },{\varOmega })$ is a weakest failure detector for consensus [12], it is possible to use classic consensus algorithms on top of this simulation. Furthermore, as ${\varSigma }$ is the weakest failure detector to simulate shared memory on top of wait-free asynchronous message passing [12], even shared memory algorithms that rely on ${\varOmega }$ could be employed.

In [5, Sec. 8], we hence investigated the potential of simulating $({\varSigma },{\varOmega })$ on top of eventually stabilizing message adversaries, as this would allow us to employ such well-established consensus solutions instead of specifically tailored algorithms. Unfortunately, it turned out that ${\varSigma }$ cannot be implemented atop many message adversaries under which consensus is solvable, including the one presented in this paper. Therefore, we concluded that, for this type of message adversaries, failure detector simulations are not a viable alternative to the approach taken here.

## Model

We consider an ensemble of deterministic state machines, called *processes*, which communicate via message passing over unreliable point-to-point links. Processes have unique identifiers and are typically denoted by $p, q, p', q',$ etc. The operation proceeds in lock-step synchronous rounds $r=1,2,3, \ldots$ consisting of a phase of message exchange between the processes, which is followed by a phase of local computations. Similar to, e.g., [17], we use the convention that all operations of round *r* take place strictly within time $r-1$ and time *r*, which results in well-defined and stable states of all processes between the rounds: The *state* of a process at time *r* is its initial state (specifying the starting values for each variable) for $r=0$, respectively the state at the end of its round-*r* computation (describing the content of all variables as well as the messages to be sent) for $r>0$. The collection of the states of all processes at time *r* is called the *configuration*$C^r$, with $C^0$ denoting the initial configuration.

A *dynamic graph* is a mapping of each round *r* to a directed graph^4^${\mathcal {G}}^r= \langle {\varPi }, E^r \rangle$, called the round-*r* communication graph. Each node of ${\varPi }$ represents a process, and an edge $({p}, {q})$ in ${\mathcal {G}}^r$ represents that the round-*r* message of *p* sent to *q* is received by *q* in the same round. Since every process *p* always successfully receives from itself, all graphs ${\mathcal {G}}^r$ are reflexive, i.e., they contain an edge $({p}, {p})$ for every process $p \in {\varPi }$. The *in-neighborhood* of *p* in ${\mathcal {G}}^r$, ${{\,\mathrm{In}\,}}_p({\mathcal {G}}^r) = \{ q \mid ({q}, {p}) \in {\mathcal {G}}^r)$ hence represents the processes from which *p* may have received a message in round *r*. We stress that the vertex set ${\varPi }$ of a given dynamic graph is fixed and only the set of edges may vary from round to round and assume that every $p \in {\varPi }$ has a unique identifier from the set $[1, |{\varPi } |]$. We often identify a dynamic graph with an infinite sequence $\sigma$ of consecutive communication graphs and denote its vertex set by ${\varPi }_\sigma$. When describing a continuous subsequence $\sigma '$ of $\sigma$, ranging from round *a* to round *b*, we denote this as $\sigma ' = ({\mathcal {G}}^{r})_{r = a}^{b}$, where $|\sigma '| = b-a+1$, with $b=\infty$ for infinite subsequences. We slightly overload notation and write $\sigma ' \subseteq \sigma$ if $\sigma '$ is a continuous subsequence of $\sigma$ and write the concatenation of two successive subsequences $\sigma '$, $\sigma ''$ as $\sigma ' \circ \sigma ''$.

A *message adversary*${{\textsf {MA}}}$ that may suppress certain messages in an attempt to foil the collaboration of the processes is at the core of our model. Formally, it is a set of dynamic graphs, or, equivalently, communication graph sequences, which are called *admissible*. Sometimes it will be convenient to denote explicitly restrictions on the size of the vertex set of the dynamic graphs of a message adversary as the first index of ${{\textsf {MA}}}$. For example, ${{\textsf {MA}}}_n$ states that every dynamic graph of ${{\textsf {MA}}}_n$ has a vertex set of size exactly *n*, while ${{\textsf {MA}}}_{\leqslant n}$ denotes that this size is at most *n*. Conceptually,^5^ we assume that processes know a priori the specification of the message adversary, hence an algorithm that succeeds under ${{\textsf {MA}}}$ must be able to cope with the size restrictions of ${{\textsf {MA}}}$. Since a message adversary is a set of dynamic graphs, we can compare different message adversaries via set inclusion.

We consider the *consensus problem*, where each process *p* starts with input value $x_p \in {\mathbb {N}}$ and has a dedicated write-once output variable $y_p$, where $y_p = \bot$ initially; eventually, every process needs to irrevocably decide, i.e., assign a value to $y_p$ (*termination*) that is the same at every process (*agreement*) and was the input of a process (*validity*). The assignment of the input values for each process is specified in the initial configuration $C^0$. Given a message adversary ${{\textsf {MA}}}$, a deterministic consensus algorithm ${\mathcal {A}}$ and a $\sigma \in {{\textsf {MA}}}$, an *admissible execution* or *run*$\varepsilon = \langle C^0, \sigma \rangle$ is a sequence of configurations $C^0, C^1 \ldots$ where for $r > 0$, $C^r$ is the result of exchanging the messages to be sent according to $C^{r-1}$ and ${\mathcal {G}}^r$, and applying the resulting state transitions specified by ${\mathcal {A}}$. Since ${\mathcal {A}}$ is deterministic, the execution $\varepsilon$ is uniquely determined by an admissible graph sequence $\sigma \in {{\textsf {MA}}}$ and a corresponding initial configuration $C^0$. Algorithm ${\mathcal {A}}$ solves consensus under message adversary ${{\textsf {MA}}}$ if, for every $\sigma \in {{\textsf {MA}}}$ and every input assignment $C^0$, validity, agreement and termination are all satisfied in the execution $\langle C^0, \sigma \rangle$ of ${\mathcal {A}}$. We will see that in some cases, the size of the set of processes ${\varPi }$ may be different in selected dynamic graphs of ${{\textsf {MA}}}$ and the processes must cope with this and the fact that they cannot reliably compute the size of ${\varPi }$. We call a consensus algorithm *uniform* (c.f. [2]) for ${{\textsf {MA}}}$ if it solves consensus under ${{\textsf {MA}}}$ and ${{\textsf {MA}}}$ consists of dynamic graphs of arbitrary size.

As usual, we write $\varepsilon \sim _{p} \varepsilon '$ if the finite or infinite executions $\varepsilon$ and $\varepsilon '$ are *indistinguishable* to *p* (i.e., the state of *p* at time *r* is the same in both executions) until *p* decides. When establishing our lower bounds, we will often exploit that, as outlined above, the configuration at time *r* is uniquely determined by the initial configuration $C^0$ and the sequence of communication graphs until round *r*.

As one of our impossibility proofs relies on a bivalence argument, we briefly rephrase the terminology from [13]: Consider an algorithm ${\mathcal {A}}$ that solves the binary consensus problem, where, for every process *p*, the initial value $x_p \in \{0, 1 \}$. Given a message adversary ${{\textsf {MA}}}$, we call a configuration $C = \langle C^0, \sigma \rangle$ of ${\mathcal {A}}$*univalent* or, more specifically, *v-valent*, if all processes decide *v* in $\langle C, \sigma ' \rangle$ for all $\sigma '$ satisfying that the concatenated sequence $\sigma \circ \sigma ' \in {{\textsf {MA}}}$. We call *Cbivalent*, if it is not univalent.

### Dynamic graph concepts

First, we introduce the pivotal notion of a *root componentR*, often called root for brevity, which denotes the vertex set of a strongly connected component of a graph where there is no edge from a process outside of *R* to a process in *R*.

#### Definition 1

(*Root component*) $R \ne \emptyset$ is a root (component) of graph ${\mathcal {G}}$, if it is the set of vertices of a strongly connected component ${{\mathcal {R}}}$ of ${\mathcal {G}}$ and $\forall p \in {\mathcal {G}}, q \in R : (p \rightarrow q) \in {\mathcal {G}}\Rightarrow p \in R$.

It is easy to see that every graph has at least one root component. A graph ${\mathcal {G}}$ that has a *single* root component is called *rooted*; its root component is denoted by ${{\,\mathrm{Root}\,}}({\mathcal {G}})$. Clearly, a graph ${\mathcal {G}}$ is rooted if and only if contracting its strongly connected components to single vertices yields a rooted tree. Hence, ${\mathcal {G}}$ contains is weakly connected and contains a directed path from every node of ${{\,\mathrm{Root}\,}}({\mathcal {G}})$ to every other node of ${\mathcal {G}}$.

Conceptually, root components have already been employed for solving consensus a long time ago: The asynchronous consensus algorithm for initially dead processes introduced in the classic FLP paper [13] relies on a suitably constructed initial clique, which is just a special case of a root component.

In order to model stability, we rely on root components that are present in every member of a (sub)sequence of communication graphs. We call such a root component the *stable root component* of a sequence and stress that, although the set of processes remains the same, the interconnection topology between the processes of the root component as well as the connection to the processes outside may vary arbitrarily from round to round.

#### Definition 2

(*Stable root component*) We say that a non-empty sequence $({\mathcal {G}}^r)_{r \in I}$ of graphs has a stable root component *R*, if and only if each ${\mathcal {G}}^r$ of the sequence is rooted and $\forall i, j \in I : {{\,\mathrm{Root}\,}}({\mathcal {G}}^i) = {{\,\mathrm{Root}\,}}({\mathcal {G}}^j) = R$. We call such a sequence an $R$-rooted sequence.

We would like to clarify that while “rooted” describes a graph property, “$R$-rooted” describes a property of a sequence of graphs.

Given two graphs ${\mathcal {G}}= \langle V, E \rangle$, ${\mathcal {G}}' = \langle V, E' \rangle$ with the same vertex set *V*, let the *compound graph*${\mathcal {G}}\circ {\mathcal {G}}' := \langle V, E'' \rangle$ where $({p}, {q}) \in E''$ if and only if there exists a $p' \in V$ such that $({p}, {p'}) \in E$ and $({p'}, {q}) \in E'$.

In order to model information propagation in the network, we use a notion of *causal past*: Intuitively, a process *q* is in *p*’s causal past, denoted $q \in {{\,\mathrm{CP}\,}}_{p}^{r}({r'})$ if, at time *r*, *p* holds information (sent either directly or transitively, via intermediate messages) that *q* sent after time $r'$. This is closely related to various concepts that have been introduced in the literature (cf. for example [7] and the references therein), such as the heard-of sets from [10] or the temporal distance from [26].

#### Definition 3

(*Causal past*) Given a sequence $\sigma$ of communication graphs that contains rounds *a* and *b*, the causal past of process *p* from time *b* down to time *a* is ${{\,\mathrm{CP}\,}}_{p}^{b}({a}) = \emptyset$ if $a \geqslant b$ and ${{\,\mathrm{CP}\,}}_{p}^{b}({a}) = {{\,\mathrm{In}\,}}_p({\mathcal {G}}^{a+1} \circ \cdots \circ {\mathcal {G}}^b)$ if $a < b$.

**Fig. 1:**
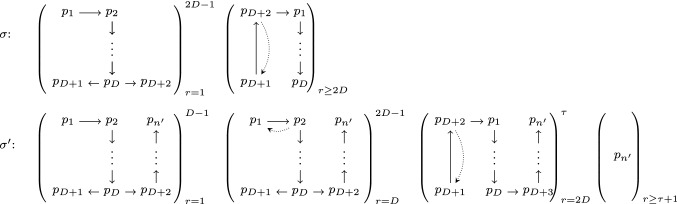
Communication graph sequences of Theorem 2, where $({\mathcal {G}})_{r=a}^b$ denotes that ${\mathcal {G}}$ is the communication graph from round *a* until round *b*. A dotted edge represents an edge which is in ${\mathcal {G}}^i$ if and only if it is not in ${\mathcal {G}}^{i-1}$. We assume that there are self-loops and there is an edge from every process depicted in the graph to every process not depicted in the graph

A useful fact about the causal past is that in full-information protocols, where processes exchange their entire state history in every round, we have $q \in {{\,\mathrm{CP}\,}}_{p}^{r}({s})$ if and only if, at time *r* (and hence thereafter), *p* knows already the state of $q\ne p$ at time *s*.

To familiarize the reader with our notation, we conclude this section with the following technical Theorem 1, which shows a structural property of our model using similar basic arguments as in [16]. It describes the information propagation in a graph sequence consisting of an ordered set $G = \left\{ {\mathcal {G}}^{r_1}, \ldots , {\mathcal {G}}^{r_n} \right\}$, $i \ne j \Rightarrow r_i \ne r_j$, and $i> j \Rightarrow r_i > r_j$, of *n* distinct communication graphs on the same vertex set ${\varPi }$, where all ${\mathcal {G}}, {\mathcal {G}}' \in G$ are rooted but ${{\,\mathrm{Root}\,}}({\mathcal {G}})$ is not necessarily the same as ${{\,\mathrm{Root}\,}}({\mathcal {G}}')$. As we have mentioned earlier, every ${\mathcal {G}}\in G$ contains a rooted spanning tree and is therefore weakly connected. In essence, the lemma shows that, by time $r_n$, each process *p* (except one process that is in the root component of ${\mathcal {G}}^{r_n}$) transitively received a message from a process *q* that was sent after *q* was member of a root component of a graph of *G*.

#### Theorem 1

Let $$G = \left\{ {\mathcal {G}}^{r_1}, \ldots , {\mathcal {G}}^{r_n} \right\} $$ be an ordered set of rooted communication graphs on the same vertex set $${\varPi }$$ where $$| {\varPi } | = n > 1$$. Pick an arbitrary mapping $$f :[1,n] \mapsto {\varPi }$$ s.t. $$f(i) \in {{\,\mathrm{Root}\,}}({\mathcal {G}}^{r_i})$$. Then $$\forall p \in {\varPi } {\setminus } \left\{ f(n) \right\} ,$$$$\exists i \in [1, n-1] :$$$$f(i) \in {{\,\mathrm{CP}\,}}_{p}^{r_n}({r_i})$$.

#### Proof

Let $S(i) = \{ p \in {\varPi } \mid \exists j \in [1, i] :p = f(j) \vee f(j) \in {{\,\mathrm{CP}\,}}_{p}^{r_i}({r_j}) \}$ be the set of nodes that, by time $r_i$, received the state of *f*(*j*) at time $r_j$ or are equal to *f*(*j*). We show by induction that $|S(n)| \geqslant n$.

The base $|S(1)| \geqslant 1$, follows because $f(1) \in S(1)$.

For the step from *i* to $i+1$, we have the hypothesis $|S(i)| \geqslant i$. Since $S(i) \subseteq S(i+1)$, we only need to consider the case $|S(i)| = i < n$. Let $p = f(i+1)$. If $p \notin S(i)$, the claim is immediate, so assume $p \in S(i)$. As $p \in {{\,\mathrm{Root}\,}}({\mathcal {G}}^{r_{i+1}})$, there is a path from *p* to every $q \in {\varPi }$ in ${\mathcal {G}}^{r_{i+1}}$. Because we assumed $|S(i)| = i < n$, there is an edge (*u*, *v*) on this path such that $u \in S(i)$ and $v \in {\varPi } {\setminus } S(i)$. By construction of *S*(*i*) and Definition 3, $v \in S(i+1)$.

It remains to be shown that $|S(n)| \geqslant n$ implies the theorem. By construction of *S*(*i*), $S(n) {\setminus } \left\{ f(n) \right\}$ contains only processes *p* for which the claim holds directly or which satisfy $p = f(j)$ for a $j \in [1, n-1]$. In case of the latter, since we assume self-loops in every communication graph, $p \in {{\,\mathrm{CP}\,}}_{p}^{r_n}({r_j})$ also holds. $\square$

## Message adversaries

First, we introduce the adversary that adheres to *dynamic network depthD*, which gives a bound on the duration of the information propagation from a stable root component to the entire network. We showed in [5, Cor. 1] that always $D\leqslant n-1$; a priori restricting $D < n-1$ also allows modelling dynamic networks where information propagation is guaranteed to be faster than in the worst case (as in expander graphs [5], for example).

### Definition 4

${{\textsf {DEPTH}}}_n(D)$ is the set of all infinite communication graph sequences $\sigma$ s.t. $|{\varPi }_\sigma |= n$ and, for all finite rounds $r_1$, for all subsequences $\sigma ' = ({\mathcal {G}}^{r_1}, \ldots , {\mathcal {G}}^{r_1+D-1})$ of $\sigma$, if $\sigma '$ is $R$-rooted, then $R \subseteq {{\,\mathrm{CP}\,}}_{p}^{r_1+D-1}({r_1-1})$ for all $p \in {\varPi }_\sigma$.

The following liveness property, *eventual stability*, ensures that eventually every graph sequence $\sigma$ has an $R$-rooted subsequence $\sigma ' \subseteq \sigma$ of length *x*. This implies that all sequences have a vertex-stable root component that consists of the same set of processes with possibly varying interconnection topology for *x* consecutive rounds.

### Definition 5

$\lozenge {{\textsf {GOOD}}}_n(x)$ is the set of all infinite communication graph sequences $\sigma$ such that $|{\varPi }_\sigma |= n$ and there exists a set $R \subseteq {\varPi }_\sigma$ and an *R*-rooted $\sigma ' \subseteq \sigma$ with $|\sigma '| \geqslant x$.

For finite *x*, $\lozenge {{\textsf {GOOD}}}_n(x)$ alone is insufficient for solving consensus: Arbitrarily long sequences of graphs that are not rooted before the stability phase occurs can fool all consensus algorithms to make wrong decisions. For this reason, we introduce a safety property in the form of the message adversary that generates only rooted graphs. As mentioned above, this implies that every communication graph is weakly connected and there is a single root component, i.e. a non-empty set of nodes from which all nodes are reachable.

### Definition 6

${{\textsf {ROOTED}}}_n$ is the set of all infinite sequences $\sigma$ of *rooted* communication graphs such that $|{\varPi }_\sigma |= n$.

The short-lived eventually stabilizing message adversary $\lozenge {{\textsf {STABLE}}}_{n,D}(D+1)$ used throughout the main part of our paper adheres to the dynamic network depth *D*, guarantees that every ${\mathcal {G}}^r$ is rooted and that every sequence has a subsequence of at least $x=D+1$ consecutive communication graphs with a stable root component. Since processes are aware under which message adversary they are executing, they have common a priori knowledge of the dynamic network depth *D* and the duration of the stability phase *x*. Moreover, depending on the variant actually used, they have some knowledge regarding the system size *n*.

### Definition 7

We call $\lozenge {{\textsf {STABLE}}}_{n,D}(x) = {{\textsf {ROOTED}}}_n\cap \lozenge {{\textsf {GOOD}}}_n(x) \cap {{\textsf {DEPTH}}}_n(D)$ the eventually stabilizing message adversary with stability period *x*. For a fixed *D*, we consider the following generalizations:

- $$\lozenge {{\textsf {STABLE}}}_{<\infty ,D}(x) = \bigcup _{n \in {\mathbb {N}} {\setminus } \left\{ 0,1 \right\} } \lozenge {{\textsf {STABLE}}}_{n,D}(x)$$
- $$\lozenge {{\textsf {STABLE}}}_{\leqslant N,D}(x) = \bigcup _{n = 2}^N \lozenge {{\textsf {STABLE}}}_{n,D}(x)$$

We observe that $\lozenge {{\textsf {GOOD}}}_n(x) \supseteq \lozenge {{\textsf {GOOD}}}_n(D)$ for all $1 \leqslant x \leqslant D$, hence it follows that $\lozenge {{\textsf {STABLE}}}_{n,D}(x) \supseteq \lozenge {{\textsf {STABLE}}}_{n,D}(D)$.

## Impossibility results and lower bounds

Even though processes know the dynamic network depth *D*, for very short stability periods, this is not enough for solving consensus. In Theorem 2, we prove that consensus is impossible under $\lozenge {{\textsf {STABLE}}}_{<\infty ,D}(2D-1)$ (recall that even if the dynamic graph has a finite set of processes ${\varPi }$, this set is not necessarily known to the processes). That is, if processes do not have access to an upper bound *N* on the number of processes, solving consensus is impossible if the period *x* of eventual stability is shorter than 2*D*: Here, processes can never be quite sure whether a stable root component occurred for at least *D* rounds, which is critical, however, since only a duration of *D* or more rounds guarantees information propagation, according to Definition 4.

The core argument of the proof is that an arbitrary correct consensus algorithm ${\mathcal {A}}$ will fail when exposed to the communication graph sequences $\sigma , \sigma '$ from Fig. 1. Fix the input values of processes $p_1, \ldots , p_{D+2}$ to 0 and let all other processes start with input 1. Because ${\mathcal {A}}$ satisfies termination, process $p_{D+1}$ eventually, by a time $\tau$, has reached a decision in an execution based on $\sigma$. Since the situation is indistinguishable for $p_{D+1}$ from the situation where everyone started with 0, it has to decide 0 by validity. Crucially, $p_{D+1}$ cannot distinguish whether the actual communication graph sequence is $\sigma$ or $\sigma '$, thus it decides 0 also in the latter. If $n'$ was chosen sufficiently large, however, process $p_{n'}$ never learns of an input value other than 1. A similar argument as above shows that, by validity, $p_{n'}$ hence eventually decides 1 and thus two values were decided under the communication graph sequence $\sigma '$. Clearly, ${\mathcal {A}}$ does not satisfy agreement, a contradiction to the initial supposition that ${\mathcal {A}}$ is correct, as $\sigma '$ is an admissible communication graph sequence.

### Theorem 2

Under $\lozenge {{\textsf {STABLE}}}_{<\infty ,D}(x)$ consensus is impossible for $0< x < 2D$.

### Proof

As we have $\lozenge {{\textsf {GOOD}}}_n(x) \subset \lozenge {{\textsf {GOOD}}}_n(x')$ for $x > x'$, it suffices to show that consensus is impossible under message adversary ${{\textsf {MA}}}= \lozenge {{\textsf {STABLE}}}_{<\infty ,D}(2D-1)$.

Pick an arbitrary $D \in {\mathbb {N}}$ and suppose an algorithm ${\mathcal {A}}$ solves consensus under ${{\textsf {MA}}}$. Let *n*, resp. $n'$, denote the number of nodes in the communication graphs of $\sigma$, resp. $\sigma '$ from Fig. 1. We provide two admissible executions $\varepsilon , \varepsilon '$ based on $\sigma$, resp. $\sigma '$ and prove that, with $\varepsilon _r, \varepsilon '_r$ denoting their first *r* rounds, for $r \leqslant \tau$ we have $\varepsilon _r \sim _{p_{D+1}} \varepsilon _r'$. We show that $p_{D+1}$ decides 0 in $\varepsilon _\tau$ and hence in $\varepsilon _\tau '$, whereas process $p_{n}$ decides 1 in $\varepsilon '$.

Let $C^0$ be the initial configuration with input values $x_p = 0$ if $p \in \{ p_1, \ldots , p_{D+2} \}$ and $x_p = 1$ otherwise, and let ${\overline{C}}^0$ be the initial configuration where for all input values we have $x_p = 0$.

Consider execution $\varepsilon = \langle C^0, \sigma \rangle$ with $\sigma$ from Fig. 1, where a dotted edge exists only in every second graph of the sequence, and all processes not depicted have an in-edge from every depicted process. We have $\sigma \in {{\textsf {MA}}}$, since it guarantees eventual stability for $2D-1$ rounds, adheres to the dynamic network depth *D* and in every round the communication graph is rooted. By the assumed correctness of ${\mathcal {A}}$, there is a finite time ${\hat{\tau }}$ by which every process has decided in $\varepsilon$; let $\tau =\max \{{\hat{\tau }},2D\}$. For $p \in \{ p_{D+1}, p_{D+2} \}$ (and by agreement thus for all processes), the decision must be 0 because we have $q \in {{\,\mathrm{CP}\,}}_{p}^{\tau }({0}) \Rightarrow x_q = 0$, hence $\varepsilon \sim _{x_p} \langle {\overline{C}}^0, \sigma \rangle$, and 0 is decided in $\langle {\overline{C}}^0, \sigma \rangle$ by validity.

Now, consider the execution $\varepsilon ' = \langle C^0, \sigma ' \rangle$ with $\sigma '$ from Fig. 1 and $n' > \tau + D + 3$. Again, $\sigma ' \in {{\textsf {MA}}}$, since $({\mathcal {G}}^{r})_{r = \tau +1}^{\infty }$ is $p_{n'}$-rooted. With $\varepsilon _r$, $\varepsilon '_r$ denoting the first *r* rounds of $\varepsilon$, resp. $\varepsilon '$, for $r \leqslant \tau$ and $p \in \{ p_{D+1}, p_{D+2} \}$, we have $\varepsilon \sim _{p} \varepsilon '$: This is immediately obvious for $1 \leqslant r \leqslant D-1$. For $D \leqslant r \leqslant \tau$, we have $\varepsilon _D \not \sim _{q} \varepsilon '_D \Leftrightarrow q = p_1$ and, as a simple induction shows, $\varepsilon _r \sim _{q} \varepsilon '_r \Leftrightarrow q \ne p_1 \wedge p_1 \notin {{\,\mathrm{CP}\,}}_{q}^{r}({D})$. It is not hard to see that $p_1 \notin {{\,\mathrm{CP}\,}}_{p}^{r}({D})$, hence $\varepsilon _r \sim _{p} \varepsilon '_r$ is maintained.

Consequently, by time $\tau$, $p_{D+1}$ has decided 0 also in $\varepsilon '$. Yet, by construction of $\varepsilon '$, for an arbitrary time *r*, we have $q \in {{\,\mathrm{CP}\,}}_{p_{n'}}^{r}({0}) \Rightarrow x_q = 1$ since $n' > \tau +D+3$. By termination, validity, and an analogous argument as above, $p_{n'}$ must hence decide 1 in $\varepsilon '$ eventually, which violates agreement and provides the required contradiction. $\square$

Theorem 2 shows that consensus is impossible under $\lozenge {{\textsf {STABLE}}}_{<\infty ,D}(D+1)$, since no process has a bound on the system size. In the remaining paper, we thus study the adversary $\lozenge {{\textsf {STABLE}}}_{\leqslant N,D}(D+1)$, for which we show in the next section that consensus can indeed be solved.

As our next result, we present a lower bound for the duration *x* of the stable period: We prove that even if there is an upper bound *N* on the number of processes in the current sequence, consensus is impossible under $\lozenge {{\textsf {STABLE}}}_{\leqslant N,D}(x)$ if $x \leqslant D$ (Theorem 3). Note that this result improves the lower bound $x \geqslant D-1$ established in [3, 5] and thus reveals that the latter was not tight. We note, however, that the proof of the earlier result is more general in that it proves bivalence when starting from an arbitrary stabilization round $r_0$; Theorem 3 shows this only for $r_0=1$, i.e., when the stable period occurs immediately.

**Fig. 2:**
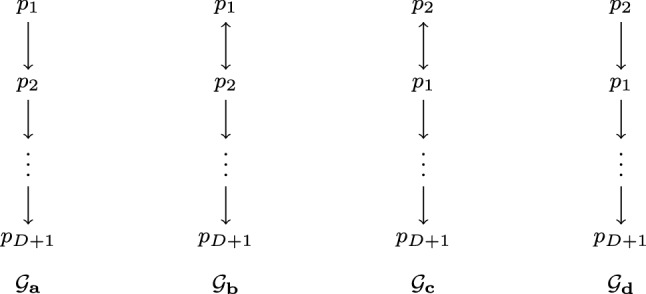
Communication graphs for Theorem 3. We assume there is an edge from every process depicted in the graph to every process not depicted in the graph

For $N=2$, our result can be derived from [22], where it was shown that consensus is impossible if at most $n-1$ messages are lost in each round. In our terminology, this means that consensus is impossible under $\lozenge {{\textsf {STABLE}}}_{\leqslant 2,1}(1)$. For general *N*, *D*, Theorem 3 below shows that a stability period of *D* or less rounds is insufficient for solving consensus for arbitrary values of $N$ as well. This result is not subsumed by [22], since the adversary is not restricted by the number of link failures but by the structure of the communication graph sequences.^6^

Informally, the reason for the impossibility is that there are executions where, even with a stability phase of *D* rounds, there is a process that cannot precisely determine the root component of the stability window. This can be seen when considering the graphs ${\mathcal {G}}_a, {\mathcal {G}}_b, {\mathcal {G}}_c, {\mathcal {G}}_d$ from Fig. 2. Fix the input values such that $x_{p_1} \ne x_{p_2}$ and, for each graph ${\mathcal {G}}_a, {\mathcal {G}}_b, {\mathcal {G}}_c, {\mathcal {G}}_d$, consider the four configurations that result when applying the graph repeatedly for *D* rounds. As these configurations are connected by an indistinguishability relation, and all processes can become the single root component “forever after” (thereby remaining unable to distinguish the four executions), not all these configurations can be univalent; if they were, the configuration resulting from applying ${\mathcal {G}}_a$ for *D* rounds would have the same valence as the one resulting from applying ${\mathcal {G}}_d$ for *D* rounds. An inductive argument, similar to the one employed by [22], shows that this bivalence can be preserved forever and hence no correct consensus algorithm exists.

Before stating our main theorem, we need to establish an essential technical lemma. It shows, for $n>2$, that by adding/removing a single edge at a time, we can arrive at a desired rooted communication graph when starting from an arbitrary other rooted communication graph. Furthermore, during this construction, we can avoid graphs that contain a certain “undesirable” root component $R''$.

### Lemma 1

Let $n>2$, let ${\mathcal {G}}$ be a rooted communication graph with ${{\,\mathrm{Root}\,}}({\mathcal {G}}) = R$, let ${\mathcal {G}}'$ be a rooted communication graph with ${{\,\mathrm{Root}\,}}({\mathcal {G}}') = R'$, and let $R''$ be a root component with $R'' \ne R$ and $R'' \ne R'$. Then, there is a sequence of communication graphs, ${\mathcal {G}}= {\mathcal {G}}_1, \ldots , {\mathcal {G}}_k = {\mathcal {G}}'$ s.t. each ${\mathcal {G}}_i$ in the sequence is rooted, ${{\,\mathrm{Root}\,}}({\mathcal {G}}_i) \ne R''$, and, for $1 \leqslant i < k$, ${\mathcal {G}}_i$ and ${\mathcal {G}}_{i+1}$ differ only in a single edge.

### Proof

Let ${\overline{{\mathcal {G}}}}$ and ${\overline{{\mathcal {G}}}}'$ be arbitrary communication graphs such that ${{\,\mathrm{Root}\,}}({\overline{{\mathcal {G}}}}) = \overline{R}$, ${{\,\mathrm{Root}\,}}({\overline{{\mathcal {G}}}}')=\overline{R}'$, and $\overline{R}'$ differs from $\overline{R}$ in at most one process, i.e., $|(\overline{R}' \cup \overline{R}) {\setminus } (\overline{R}' \cap \overline{R}) |\leqslant 1$. We show that there is a sequence ${\overline{{\mathcal {G}}}}= {\overline{{\mathcal {G}}}}_1, \ldots , {\overline{{\mathcal {G}}}}_j = {\overline{{\mathcal {G}}}}'$ such that each ${\overline{{\mathcal {G}}}}_i$ in the sequence is rooted with ${{\,\mathrm{Root}\,}}({\overline{{\mathcal {G}}}}_i) \in \{ \overline{R}, \overline{R}' \}$ and, for $1 \le i < j$, ${\overline{{\mathcal {G}}}}_i$ differs from ${\overline{{\mathcal {G}}}}_{i+1}$ in a single edge. Repeated application of this fact implies the lemma, because for $n>2$ we can always find a sequence $R = R_1, \ldots , R_l = R'$ of subsets of ${\varPi }$ s.t. for each $R_i$ of the sequence we have $R_i \ne R''$ and, for $1 \leqslant i < l$, $R_i$ differs from $R_{i+1}$ by exactly one process. To see this, we observe that in the Hasse diagram of the power set of ${\varPi }$, ordered by set inclusion, there are always two upstream paths leading from two aribtrarily chosen subsets of ${\varPi }$ to a common ancestor.

We now sketch how to construct the desired communication graphs ${\overline{{\mathcal {G}}}}_i$ of the sequence in two phases:

*Phase 1a:* If we need to add a node *p* to $\overline{R}= {{\,\mathrm{Root}\,}}({\mathcal {G}}_i)$ to arrive at $\overline{R}'$, for a $q \in \overline{R}$, first add $(q \rightarrow p)$. For all $q' \notin \{p, q \}$ remove $(q' \rightarrow p)$. Finally, add $(p \rightarrow q)$.

*Phase 1b:* If we need to remove a node *p* from $\overline{R}$ to arrive at $\overline{R}'$, first add edges (one by one) between nodes of $\overline{R}$ until the nodes of $\overline{R}$ are completely connected. Then, iteratively remove all edges $(p \rightarrow q)$ with $q \in \overline{R}'$.

*Phase 2:* Since we now already have a communication graph ${\overline{{\mathcal {G}}}}_i$ with ${{\,\mathrm{Root}\,}}({\overline{{\mathcal {G}}}}_i) = \overline{R}'$, it is easy to add/remove edges one by one to arrive at the topology of ${\overline{{\mathcal {G}}}}'$. First, we add edges until the nodes of $\overline{R}'$ are completely connected among each other, the nodes not in $\overline{R}'$ are completely connected among each other, and there is an edge from every node of $\overline{R}'$ to each node not in $\overline{R}'$. Second, we remove the edges not present in ${\overline{{\mathcal {G}}}}'$. $\square$

### Theorem 3

There is no consensus algorithm for $\lozenge {{\textsf {STABLE}}}_{\leqslant N,D}(x)$ with $1 \leqslant x \leqslant D$, even if the adversary guarantees that the first *D* rounds are $R$-rooted.

### Proof

In the case where $D=1$, we need to show the impossibility of $\lozenge {{\textsf {STABLE}}}_{\leqslant N,D}(1)$. We immediately note that $\sigma \in \lozenge {{\textsf {STABLE}}}_{\leqslant N,D}(1)$ if and only if each ${\mathcal {G}}\in \sigma$ is rooted and a has graph diameter of 1. Clearly, the graph sequence where (i) in every graph ${\mathcal {G}}$, two fixed processes *p*, *q* have non-self-loop in-edges at most from each other and $(p, q) \in {\mathcal {G}}\vee (q, p) \in {\mathcal {G}}$, and (ii) all other processes have an in-edge from both *p* and *q* is in $\lozenge {{\textsf {STABLE}}}_{\leqslant N,D}(1)$. This, however, can be reduced to solving consensus among the two processes *p*, *q* with up to one link failure between them, which was shown to be impossible in [22].

For the remainder of the proof, let us thus assume $D \geqslant 2$. Since $\lozenge {{\textsf {STABLE}}}_{\leqslant N,D}(x) \supset \lozenge {{\textsf {STABLE}}}_{\leqslant N,D}(D)$ for $x \leqslant D$, it suffices to show the impossibility of consensus under $\lozenge {{\textsf {STABLE}}}_{\leqslant N,D}(D)$: If the execution $\varepsilon$ where consensus cannot be solved is admissible under message adversary $\lozenge {{\textsf {STABLE}}}_{\leqslant N,D}(D)$, it is still admissible under $\lozenge {{\textsf {STABLE}}}_{\leqslant N,D}(x)$. The proof proceeds roughly along the lines of [23, Lemma 3]. It first shows that for all consensus algorithms there is a bivalent configuration at time *D* and proceeds to show by induction that every bivalent configuration has a bivalent successor configuration. Hence, all consensus algorithms permit a perpetually bivalent execution under $\lozenge {{\textsf {STABLE}}}_{\leqslant N,D}(D)$, where consensus cannot be solved.

We show that a bivalent execution is even contained in $\lozenge {{\textsf {STABLE}}}_{\leqslant N,D}'(D)\subseteq \lozenge {{\textsf {STABLE}}}_{\leqslant N,D}(D)$, which consists of those executions of $\lozenge {{\textsf {STABLE}}}_{\leqslant N,D}(D)$ where already the first *D* rounds are $R$-rooted.

For the induction base, we show that not all configurations of ${\mathcal {A}}$ at time *D* can be univalent: Assume that an algorithm ${\mathcal {A}}$ solves consensus under $\lozenge {{\textsf {STABLE}}}_{\leqslant N,D}'(D)$ and suppose that all configurations of ${\mathcal {A}}$ at time *D* were univalent.

Let $C^0$ be an initial configuration of ${\mathcal {A}}$ with $x_{p_1} = 0$ and $x_{p_2} = 1$ and recall the graphs ${\mathcal {G}}_a, {\mathcal {G}}_b, {\mathcal {G}}_c$ and ${\mathcal {G}}_d$ from Fig. 2. For $i \in \{a, b, c, d \}$ let $C^D_i = \langle C^0, ({\mathcal {G}}_i)_{r= 1}^{D} \rangle$ denote the configuration which results from applying ${\mathcal {G}}_i$*D* times to $C^0$. Let ${\mathcal {S}}({p})$ denote the star-like graph where there is an edge from the center vertex *p* to every other vertex and from every vertex to itself but there are no other edges in the graph. Clearly, $C^D_a$ is 0-valent since $\langle C^D_a, ({\mathcal {S}}({p_1}))_{D+1}^{\infty } \rangle \in \lozenge {{\textsf {STABLE}}}_{\leqslant N,D}'(D)$ and for $p_1$ this is indistinguishable from the situation where all processes *p* have $x_p = 0$. A similar argument shows that $C^D_d$ is 1-valent.

Consider two cases:

1. $C^D_b$ is 1-valent. But then, $C^D_a$ cannot be 0-valent since $\langle C^D_a, ({\mathcal {S}}({p_{D+1}}))_{D+1}^{\infty } \rangle \sim _{p_{D+1}} \langle C^D_b, ({\mathcal {S}}({p_{D+1}}))_{D+1}^{\infty } \rangle$.
2. $C^D_b$ is 0-valent. Then, since $\langle C^D_b, ({\mathcal {S}}({p_{1}}))_{D+1}^{\infty } \rangle \sim _{p_{1}} \langle C^D_c, ({\mathcal {S}}({p_{1}}))_{D+1}^{\infty } \rangle$, $C^D_c$ is also 0-valent. But then $C^D_d$ cannot be 1-valent because $\langle C^D_c, ({\mathcal {S}}({p_{D+1}}))_{D+1}^{\infty } \rangle \sim _{p_{D+1}} \langle C^D_d, ({\mathcal {S}}({p_{D+1}}))_{D+1}^{\infty } \rangle$.

Hence, not all configurations at time *D* are univalent.

For the induction step, let us assume that there exists a bivalent configuration $C^r$ for a time $r \geqslant D$. For a contradiction, assume that all configurations at time $(r+1)$ reachable from $C^r$ are univalent. Thus, there exists a 0-valent configuration $C_0^{r+1} = \langle C^r, {\mathcal {G}}_0 \rangle$ that results from applying a communication graph ${\mathcal {G}}_0$ to $C^r$. Moreover, there is a 1-valent configuration $C_1^{r+1} = \langle C^r, {\mathcal {G}}_1 \rangle$ that results from applying a communication graph ${\mathcal {G}}_1$ to $C^r$.

First, let us show that for ${\mathcal {G}}\in \{ {\mathcal {G}}_0, {\mathcal {G}}_1 \}$, it holds that, if ${{\,\mathrm{Root}\,}}({\mathcal {G}}) = {{\,\mathrm{Root}\,}}({\mathcal {G}}^r)$, there is an applicable graph ${\mathcal {G}}'$ s.t. $\langle C^r, {\mathcal {G}}' \rangle$ has the same valency as $\langle C^r, {\mathcal {G}} \rangle$ and ${{\,\mathrm{Root}\,}}({\mathcal {G}}) \ne {{\,\mathrm{Root}\,}}({\mathcal {G}}')$. The reason for this is that we can construct ${\mathcal {G}}'$ from ${\mathcal {G}}$ by simply adding an edge $(p \rightarrow q)$ for a $q \ne p$, $p \not \in {{\,\mathrm{Root}\,}}({\mathcal {G}})$, $q \in {{\,\mathrm{Root}\,}}({\mathcal {G}})$ if $|{{\,\mathrm{Root}\,}}({\mathcal {G}})| = 1$, respectively, by removing $(p \rightarrow q)$ for a $p \in {{\,\mathrm{Root}\,}}({\mathcal {G}})$ and all $p \ne q \in {{\,\mathrm{Root}\,}}({\mathcal {G}})$ if $|{{\,\mathrm{Root}\,}}({\mathcal {G}})| > 1$. This yields a graph ${\mathcal {G}}'$ with the desired property, as $\langle C^r, {\mathcal {G}}, ({\mathcal {S}}({p}))_{r+1}^{\infty } \rangle \sim _{p} \langle C^r, {\mathcal {G}}', ({\mathcal {S}}({p}))_{r+1}^{\infty } \rangle$. The applicability of ${\mathcal {G}}'$ follows because ${\mathcal {G}}'$ is rooted and ${{\,\mathrm{Root}\,}}({\mathcal {G}}') \ne {{\,\mathrm{Root}\,}}({\mathcal {G}}^r)$ ensures that the resulting subsequence is a prefix of a sequence of ${{\textsf {DEPTH}}}_n(D)$ for all $D>1$, because, for these choices of *D*, a changing root component trivially satisfies Definition 4.

Hence there are graphs ${\mathcal {G}}'_0, {\mathcal {G}}'_1$ such that ${{\,\mathrm{Root}\,}}({\mathcal {G}}'_0) \ne {{\,\mathrm{Root}\,}}({\mathcal {G}}^r)$, ${{\,\mathrm{Root}\,}}({\mathcal {G}}'_1) \ne {{\,\mathrm{Root}\,}}({\mathcal {G}}^r)$, and $\langle C^r, {\mathcal {G}}'_0 \rangle$ is 0-valent while $\langle C^r, {\mathcal {G}}'_1 \rangle$ is 1-valent. As we assumed $D \geqslant 2$ it follows that $n > 2$. We can hence apply Lemma 1 to go from ${\mathcal {G}}'_0$ to ${\mathcal {G}}'_1$ by adding/removing a single edge at a time, without ever arriving at a graph that has more than one root component or has the same root component as ${\mathcal {G}}^r$. Somewhere during adding/removing a single edge, we transition from a graph ${\mathcal {G}}_i$ to a graph ${\mathcal {G}}_{i+1}$, by modifying an edge $(p \rightarrow q)$, where the valency of $C = \langle C^r, {\mathcal {G}}_i \rangle$ differs from the valency of $C' = \langle C^r, {\mathcal {G}}_{i+1} \rangle$. Nevertheless, ${\mathcal {G}}_i$ and ${\mathcal {G}}_{i+1}$, are applicable to $C^r$ because they are rooted and have a different root component as ${\mathcal {G}}^r$, hence guarantee the membership of the sequence in ${{\textsf {DEPTH}}}_n(D)$ for all $D > 1$. However, *C* and $C'$ cannot have a different valency because $\langle C, ({\mathcal {S}}({p}))_{r+1}^{\infty } \rangle \sim _{p} \langle C', ({\mathcal {S}}({p}))_{r+1}^{\infty } \rangle$. This is a contradiction and hence not all configurations at time $(r+1)$ can be univalent. $\square$

## Solving consensus with $D+1$ rounds of stability

We now present our consensus algorithm for the message adversary $\lozenge {{\textsf {STABLE}}}_{\leqslant N,D}(D+1)$, where a bound $N \geqslant n$ is known a priori. The pseudo-code for the main algorithm is presented in Algorithm 2. It relies on a collection of functions given in Algorithm 1.

Since the detailed correctness proof is somewhat tedious, we first give an informal description of the algorithm where we provide references to the lemmas that correspond to our informal description.
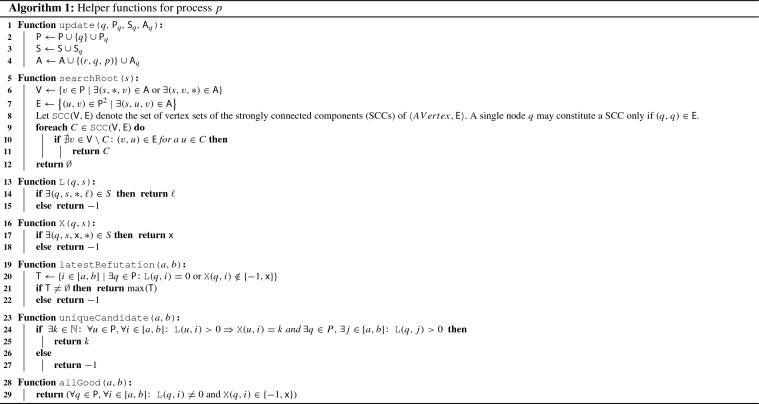

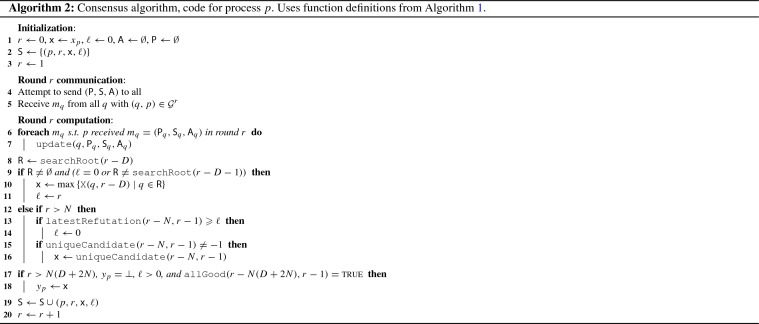


*Overview* In essence, each process *p* that executes Algorithm 2 tries to solve consensus by approximating the current graph sequence and keeping track of the relevant partial states of the other processes. Most prominently, this includes their proposal value $\textsf {x}$, basically their current decision value estimate. If a process observes that in the current graph sequence, the stability phase could have occurred, i.e., it finds a root component *R* that might have been stable for $D+1$ rounds, it locks on to the maximum over the proposal values of the members of *R*. Subsequently, *p* waits if there is evidence refuting its observation. As soon as *p* finds such contradictory evidence, it clears its locked-on state. If, on the other hand, *p* does not find such evidence for a sufficiently long time, it decides on its proposal value. In order for the latter to be a safe decision, the algorithm has a mechanism that guarantees the following: As soon as a process *q* detects that there might have been a process *p* that is convinced it holds the correct proposal value, *q* adopts the proposal value of *p*. Crucially, *q* does not enter a locked-on state in this case. The main difficulties of this approach, apart from implementing the usual graph approximation and book-keeping mechanisms, are to ensure that (1) it cannot happen that distinct processes are simultaneously convinced of two different proposal values for too long, that (2) the significant information propagation delays, inherent in this setting, still guarantee a timely adaptation of the proposal of a convinced process in the entire system, while maintaining that (3) the stability period will eventually lead to the same decision at every process.

*Detailed description* The essential data structures that each process *p* maintains are the set of the (reduced) *past process states*$\textsf {S}$, the *communication graphs approximation*$\textsf {A}$ of the communication graphs that occurred so far, and the *set of participating processes*$\textsf {P}$ of whose existence *p* learned by now (Lemma 3; recall that, given a $\sigma \in \lozenge {{\textsf {STABLE}}}_{\leqslant N,D}(D+1)$, *p* does not have exact knowledge of $|{\varPi }_\sigma |$ and hence no a priori knowledge of ${\varPi }_\sigma$). Process *p* also holds two crucial local variables, the *lock round*$\ell$ and the *proposal value*$\textsf {x}$, whose significance we outline below. In order to access $\textsf {S}$ in a clear way, Algorithm 1 provides the functions $\textsf {L}(q,s)$ and $\textsf {X}(q,s)$ that, when executed by *p*, return the value of $\ell$ resp. $\textsf {x}$ of remote process *q* at time *s*, or $-1$ if this value is unknown to *p* (Lemma 4). At the start of each round *r*, process *p* attempts to broadcast $\textsf {P}$, $\textsf {S}$, and $\textsf {A}$. By our system model, every process *q* with $(p, q) \in {\mathcal {G}}^r$ will receive this message. Maintenance of $\textsf {S}$ and $\textsf {A}$ is delegated primarily to the $\textsf {update}$ function, which merges the content of all messages received from a process *q* into the local copies of $\textsf {A}$ and $\textsf {S}$ and adds an edge (*q*, *p*), labeled with the appropriate round, to $\textsf {A}$. In addition to this, *p* appends its own value of $\ell$ and $\textsf {x}$ to $\textsf {S}$ at the end of round *r*.

Before we continue with the logic of the main algorithm, we note a key insight about our system model: To some extent, by recording received messages, interpreting them as in-edges in a communication graph, and trying to flood the system with these records, processes can reliably detect root components! Algorithm 1 implements this in the function $\textsf {searchRoot}(s)$, which, when called by *p* in round *r*, returns a set *R* that satisfies two key properties: If $R \ne \emptyset$ then $R = {{\,\mathrm{Root}\,}}({\mathcal {G}}^s)$ (Lemma 6) and if $R = {{\,\mathrm{Root}\,}}({\mathcal {G}}^s)$ and there is a chain of messages such that $R \subseteq {{\,\mathrm{CP}\,}}_{p}^{r}({s})$ then $\textsf {searchRoot}(s) = R$ (Lemma 5).

The very basic operation of Algorithm 2 is to find root components and “lock on” to them, by setting the lock round $\ell$ to the current round and $\textsf {x}$ to a deterministic function (e.g. $\max ()$) of the proposal values of the root members. In some sense, the process hopes to hit $\rho$ of the $\rho$-rooted sequence of length $D+1$ that is promised by the specification of $\lozenge {{\textsf {STABLE}}}_{\leqslant N,D}(D+1)$. After this, a process simply waits for contradictory evidence that suggests that the currently locked-on root component could not have been $\rho$. In more detail, in each round *r*, Algorithm 2 proceeds in two phases:

In the first phase, *p* checks whether it needs to adapt its values of $\textsf {x}$ or $\ell$. It does this in three cases:

1. When *p* detects that a root component *R* occurred *D* rounds ago and *p* either had $\ell = 0$ or, $D+1$ rounds ago, had detected a different root component $R' \ne R$. In either case, *p* sets its lock round $\ell \leftarrow r$ and its proposal $\textsf {x}$ gets the maximum proposal $\textsf {x}_q$ value over all processes $q \in R$ from *D* rounds ago in Line 9 (Lemma 11). This works because if the root component $R'$ that was found was indeed $\rho$, every process must have locked on to it and thus, when a process detects a changed root component *R*, it is guaranteed that all members of *R* are already locked-on to $\rho$. In this way the proposal value of $\rho$ is preserved forever (Lemma 15).
2. If the detection from the previous case failed, *p* sets $\ell \leftarrow 0$ if there is evidence that contradicts the current proposal value $\textsf {x}$ and lock round $\ell$. That is, if a process *q* had $\ell _q = 0$ or a proposal different from $\textsf {x}$ within the last *N* rounds but after the current lock round. Algorithm 1 implements this in the function $\textsf {latestRefutation}(r-N, r-1)$ (Lemma 9), as called in Line 13. The main aspect of this procedure is that *p* cannot possibly remove a lock on $\rho$, as $\rho$ would lead all processes to lock on to it and remain locked on to it forever, hence there is no contradictory evidence (Lemma 15).
3. Possibly in addition to case 2 above, process *p* adapts its own proposal to *v* if *p* sees that every process that was locked on to something during the last *N* rounds was locked on to *v*. This is to ensure that processes adapt their proposal if there is a set of locked-on processes that never learned of contradictory evidence and might be tempted to assume that their lock value stems from $\rho$ itself. In this case, the function $\textsf {uniqueCandidate}(r-N, r-1)$ returns the value *v* when called in Line 16 (Lemma 7 and Lemma 8).

In the second phase, process *p* waits until it is safe to decide on $\textsf {x}$. This is the case when, according *p*’s point of view, in the last $N(D+2N)$ rounds all processes are locked on and have the same proposal value. Process *p* performs this check via the call to $\textsf {allGood}(r-N(D+2N), r-1)$ in Line 17 (Lemma 10). The crucial point here is that, by a pigeon-hole argument, the observation of $N(D+2N)$ rounds where all processes are locked on and have the same proposal value implies that there was in fact a “*v*-locked sequence” of $D+2N$ rounds (Lemma 13). A *v*-locked sequence (Definition 8) consists only of communication graphs where every process in the root component is locked on and has the same proposal value. Such a sequence guarantees that all future proposal values are *v* (Lemma 12), thereby ensuring the safety of the algorithm.

### Correctness proof

We now prove the correctness of Algorithm 2 under an arbitrary $\sigma \in \lozenge {{\textsf {STABLE}}}_{\leqslant N,D}(D+1)$. For variable $\textsf {v}$, in our analysis, we use $\textsf {v}_p^r$ to denote the value of $\textsf {v}$ at process *p* at time *r* (or, equivalently, the value that $\textsf {v}$ had after it was written to by *p* for the last time in round *r*). Similarly, we use $\textsf {func}_p^r(\textsf {v})$ to describe the return value of function $\textsf {func}$ when called by *p* with parameter $\textsf {v}$ during its round-*r* computation. Since Algorithm 2 calls functions that provide a return value only on Lines 8–18 and these functions rely exclusively on $\textsf {P}$, $\textsf {S}$, and $\textsf {A}$, neither of which is modified in Lines 8–18, this is equivalent to their return value from Line 8 onwards but before executing Line 18.

#### Algorithm 1: Supporting functions

Before commencing with the proof that our algorithm satisfies the consensus specification, we show some essential properties of the supporting functions in Algorithm 1. As a preliminary, Lemma 2 shows that $\textsf {A}_p^r$ contains an under-approximation of the edges of ${\mathcal {G}}^s$ and that, if *p* learned that $(u, v) \in {\mathcal {G}}^s$, then *p* learned all the in-edges of *v* in ${\mathcal {G}}^s$ as well. The following Lemma 3 and Lemma 4 establish the connection between the causal past, the value of $\textsf {P}$ and the return values of the functions $\textsf {L}()$ and $\textsf {X}()$.

##### Lemma 2

Pick an arbitrary time *s* and let $r > s$. $(s, u, v) \in \textsf {A}_p^r$ if and only if $(u, v) \in {\mathcal {G}}^s$ and $v \in {{\,\mathrm{CP}\,}}_{p}^{r}({s})$.

##### Proof

First, we show that $(s, u, v) \in \textsf {A}_p^r$ implies $(u, v) \in {\mathcal {G}}^s$. By Line 4 of Algorithm 1, at time *s*, $(s, u, v) \in \textsf {A}_w^s$ if and only if $w = v$. Hence *v* called Line 7 of Algorithm 2 in round *s* as it received a message from *u* in that round. But then $(u, v) \in {\mathcal {G}}^s$, as per our system model.

Next, we show by induction on $r\geqslant s+1$ that $(s, u, v) \in \textsf {A}_p^r$ implies $v \in {{\,\mathrm{CP}\,}}_{p}^{r}({s})$.

For $r = s+1$, if $v = p$, the claim is immediate. If $v \ne p$, we have again that $(r-1, u, v) \in \textsf {A}_w^{r-1}$ if and only if $v = w$. Because we assume $(r-1, u, v) \in \textsf {A}_p^r$, there must be $(v, p) \in {\mathcal {G}}^{r}$, which implies $v \in {{\,\mathrm{CP}\,}}_{p}^{r}({r-1})$ by Definition 3.

For every time $r > s+1$, if $(s, u, v) \in \textsf {A}_p^{r-1}$, as we assume self-loops in every communication graph, the claim follows from the induction hypothesis and Definition 3. So, let $(s, u, v) \notin \textsf {A}_p^{r-1}$. Since we assume $(s, u, v) \in \textsf {A}_p^r$, this means that $(s, u, v) \in \textsf {A}_q^{r-1}$ for a $q \in {\varPi }_\sigma$ with $(q, p) \in {\mathcal {G}}^r$. Applying the hypothesis to *q* yields $v \in {{\,\mathrm{CP}\,}}_{q}^{r-1}({s})$. Taken together, this means that $v \in {{\,\mathrm{CP}\,}}_{p}^{r}({s})$ by Definition 3.

Finally, we show by induction on $r\geqslant s+1$ that $(u, v) \in {\mathcal {G}}^s$ and $v \in {{\,\mathrm{CP}\,}}_{p}^{r}({s})$ implies $(s, u, v) \in \textsf {A}_p^r$.

For $r = s+1$, because $(u,v) \in {\mathcal {G}}^{r-1}$, Line 4 of Algorithm 1 adds $(r-1, u, v)$ to $\textsf {A}_v^{r-1}$. Since $v \in {{\,\mathrm{CP}\,}}_{p}^{r}({r-1})$, *p* receives $\textsf {A}_v^{r-1}$ in a round-*r* message from *v* and adds it to $\textsf {A}_p^r$.

For $r > s+1$, the assumption that $v \in {{\,\mathrm{CP}\,}}_{p}^{r}({s})$ allows us to use Definition 3 to find a $q \in {\varPi }_\sigma$ such that $v \in {{\,\mathrm{CP}\,}}_{q}^{r-1}({s})$ and $(q, p) \in {\mathcal {G}}^r$. As we assume $(u, v) \in {\mathcal {G}}^s$, the hypothesis asserts that $(s, u, v) \in \textsf {A}_q^{r-1}$. Hence *p* adds (*s*, *u*, *v*) to $\textsf {A}_p^r$ in Line 4 of Algorithm 1 when it receives $\textsf {A}_q^{r-1}$ with the round-*r* message of *q*. $\square$

##### Lemma 3

If $$q \in {{\,\mathrm{CP}\,}}_{p}^{r}({1})$$ then $$q \in \textsf {P}_p^r$$.

##### Proof

By induction on $r\geqslant 1$. The base case, $r=1$, holds because, by Definition 3, ${{\,\mathrm{CP}\,}}_{p}^{1}({1}) = \emptyset$. For $r>1$, suppose $q \in {{\,\mathrm{CP}\,}}_{p}^{r}({1})$ and $q \notin \textsf {P}_p^r$. As $\textsf {P}_p^r$ never shrinks ($\textsf {P}$ is only modified in Algorithm 1, Line 2), $q \notin \textsf {P}_p^{r-1}$. By the hypothesis thus $q \notin {{\,\mathrm{CP}\,}}_{p}^{r-1}({1})$. But since $q \in {{\,\mathrm{CP}\,}}_{p}^{r}({1})$, by Definition 3, either $q\in {{\,\mathrm{In}\,}}_p({\mathcal {G}}^r)$ or else there is a process *u* s.t. $q \in {{\,\mathrm{CP}\,}}_{u}^{r-1}({1})$ and $u \in {{\,\mathrm{In}\,}}_p({\mathcal {G}}^r)$. In the latter case, invoking the hypothesis for *u* reveals that $q \in \textsf {P}_{u}^{r-1}$. In both cases, *q* is added by Line 2 of Algorithm 1 during the round-*r* computation of process *p*. Hence $q \in \textsf {P}_p^r$, a contradiction. $\square$

##### Lemma 4

$\textsf {L}_p^r(q, s)$ returns $\ell _q^s$ if $q \in {{\,\mathrm{CP}\,}}_{p}^{r}({s})$ and $-1$ otherwise. Similarly, $\textsf {X}_p^r(q, s)$ returns $\textsf {x}_q^s$ if $q \in {{\,\mathrm{CP}\,}}_{p}^{r}({s})$ and $-1$ otherwise.

##### Proof

We show the claim for $\textsf {L}_p^r(q,s)$. The proof for $\textsf {X}_p^r(q,s)$ is analogous. We proceed by induction on $r\geqslant s$.

For $r=s$, we observe that *p* cannot receive a message containing $(q, s, x_q^s, \ell _q^s)$ from a process *q* before round $s+1$. Hence $(q, s, x_q^s, \ell _q^s) \notin \textsf {S}_p^s$ and $\textsf {L}_p^s(q,s)$ returns $-1$, which shows the claim as $q \notin {{\,\mathrm{CP}\,}}_{p}^{s}({s})$ because ${{\,\mathrm{CP}\,}}_{p}^{s}({s}) = \emptyset$ according to Definition 3.

For $r > s$, we distinguish two cases (i) and (ii):

(i) $q \notin {{\,\mathrm{CP}\,}}_{p}^{r}({s})$. By the induction hypothesis and Definition 3, every $u \in {{\,\mathrm{In}\,}}_p({\mathcal {G}}^r)$ (including *p* itself) satisfy $u \ne q$ and $\textsf {L}_u^{r-1}(q, s) = -1$. Due to Line 14 of Algorithm 1, $\not \exists (q,s,*, *) \in \textsf {S}_u^{r-1}$. Thus, when calling $\textsf {update}(u, \textsf {P}_u, \textsf {S}_u, \textsf {A}_u)$ in round *r*, no tuple $(q, s, *, *)$ is added to $\textsf {A}_p$ and thus $\textsf {L}_p^r(q, s) = -1$.

(ii) $q \in {{\,\mathrm{CP}\,}}_{p}^{r}({s})$. Suppose $\textsf {L}_p^r(q, s) \ne \ell _q^s$. Since every tuple $(q, s, *, \ell )$ of $\textsf {S}_p^r$ originated at process *q* in round *s* at Line 19 of Algorithm 2, we have $\ell = \ell _q^s$. This implies that $\not \exists (q, s, *, *) \in \textsf {S}_p^r$. As $\textsf {S}_p^r$ never shrinks, $\not \exists (q, s, *, *) \in \textsf {S}_p^{r-1}$ and hence $\textsf {L}_p^{r-1}(q, s) = -1$. By the induction hypothesis, $q \notin {{\,\mathrm{CP}\,}}_{p}^{r-1}({s})$. By the assumption that $q \in {{\,\mathrm{CP}\,}}_{p}^{r}({s})$ and Definition 3, either $q\in {{\,\mathrm{In}\,}}_p({\mathcal {G}}^r)$ or there is a process $u \in {{\,\mathrm{In}\,}}_p({\mathcal {G}}^r)$ with $q \in {{\,\mathrm{CP}\,}}_{u}^{r-1}({s})$. In the former case, we set $u=q$, in the latter case, applying the induction hypothesis to *u* reveals $(q, s, *, \ell _q^s) \in \textsf {S}_u^{r-1}$. In both cases, *p* calls $\textsf {update}(u, *, \textsf {S}^{r-1}_u, *)$ in Line 7 in round *r* and thus adds $\textsf {S}^{r-1}_u$ to $\textsf {S}^r_p$ in Line 3 of Algorithm 1. Hence $(u, s, *, \ell _q^s) \in \textsf {S}^r_p$ and, by Line 14 of Algorithm 1, $\textsf {L}_p^r(q, s) = \ell _q^s$, a contradiction. $\square$

The following Lemmas 5 and 6 prove the formal properties the function $\textsf {searchRoot}()$ provides for the main consensus algorithm. In our analysis, we use $\textsf {E}_p^r$, $\textsf {V}_p^r$ to denote the value of $\textsf {E}$, $\textsf {V}$ at process *p* after *p* finished Line 7 of Algorithm 1 during a call of $\textsf {searchRoot}^r_p(s)$.

##### Lemma 5

Pick $\sigma \in \lozenge {{\textsf {STABLE}}}_{\leqslant N,D}(D+1)$, fix a round *s* and let $r > s$. If either of the following hold:

1. $$R = {{\,\mathrm{Root}\,}}({\mathcal {G}}^{s})$$ and $$R \subseteq {{\,\mathrm{CP}\,}}_{p}^{r}({s})$$, or
2. $({\mathcal {G}}^{i})_{i = s}^{s+D}$ is $R$-rooted and $r \geqslant s+D$,

then $\textsf {searchRoot}^{r}_p(s) = R$ for every process *p*.

##### Proof

Since $\sigma \in \lozenge {{\textsf {STABLE}}}_{\leqslant N,D}(D+1)$ we have $\sigma \in {{\textsf {DEPTH}}}_n(D)$ with $n \leqslant N$ and, by Definition 4, for all $p \in {\varPi }_\sigma$ and all $q \in R$ we have $q \in {{\,\mathrm{CP}\,}}_{p}^{r}({s})$ if (1) or (2) holds. Since furthermore $R = {{\,\mathrm{Root}\,}}({\mathcal {G}}^{s})$, by Lemma 2 and Lines 6 and 7 of Algorithm 1, *R* is a root component of $\left\langle \textsf {V}_p^r, \textsf {E}_p^r \right\rangle$ after *p* finished Line 7 during a call of $\textsf {searchRoot}_p^{r}(s)$.

If *R* is the unique root component of $\left\langle \textsf {V}_p^r, \textsf {E}_p^r \right\rangle$, then the loop of Line 9 in Algorithm 1 returns *R* as *R* is the only strongly connected component of $\left\langle \textsf {V}_p^r, \textsf {E}_p^r \right\rangle$ that has no incoming edge from a node not in *R*.

If *R* is not the unique root component of $$\left\langle \textsf {V}_p^r, \textsf {E}_p^r \right\rangle $$, then $${\mathcal {G}}^{s}$$ cannot be rooted by Lemma 2. But then $$\not \exists n \leqslant N :$$$$\sigma \in {{\textsf {ROOTED}}}_n$$, contradicting $$\sigma \in \lozenge {{\textsf {STABLE}}}_{\leqslant N,D}(D+1)$$. $$\square $$

##### Lemma 6

$$\forall r > s$$$$\forall p \in {\varPi }_\sigma :$$ if $$R = \textsf {searchRoot}^r_p(s) \ne \emptyset $$, then

1. $$R = {{\,\mathrm{Root}\,}}({\mathcal {G}}^s)$$,
2. $$\forall q \in R :$$$$q \in {{\,\mathrm{CP}\,}}_{p}^{r}({s})$$, and
3. if $$\exists t \leqslant s :$$$${{\,\mathrm{Root}\,}}({\mathcal {G}}^t) = R$$ then $$\textsf {searchRoot}^r_p(t) = R$$.

##### Proof

To show the lemma, let us assume that $R = \textsf {searchRoot}_p^r(p,s) \ne \emptyset$, i.e., according to the loop of Line 9 in Algorithm 1, for all $u \in R$, $v \in \textsf {V}_p^r {\setminus } R$, there is no edge $(v,u) \in \textsf {E}_p^r$

(1) Since *R* was returned by the loop in Line 9 of Algorithm 1, *R* is a strongly connected component of $\left\langle \textsf {V}_p^r, \textsf {E}_p^r \right\rangle$, which implies that for every *v* of *R* there is an edge $(*, v) \in \textsf {E}_p^r$. By Lemma 2, if *p* learned one in-edge of a node *v* in ${\mathcal {G}}^s$, it learned all in-edges of *v* in ${\mathcal {G}}^s$. Thus, *R* is also a strongly connected component in ${\mathcal {G}}^s$, and there is no edge in ${\mathcal {G}}^s$ to a node of *R* from a node not in *R*. Taken together, this matches precisely Definition 1, hence $R = {{\,\mathrm{Root}\,}}({\mathcal {G}}^s)$.

(2) By the construction of $\left\langle \textsf {V}_p^r, \textsf {E}_p^r \right\rangle$ in Lines 6 and 7 of Algorithm 1, an edge $(u, v) \in \textsf {E}_p^r$ if and only if $(s, u, v) \in \textsf {A}^r_p$. As we have shown above, *R* is the root component of $\left\langle \textsf {V}_p^r, \textsf {E}_p^r \right\rangle$. Therefore, for all $v \in R$ there is an edge $(s, u, v) \in \textsf {A}_p^r$. By Lemma 2, $v \in {{\,\mathrm{CP}\,}}_{p}^{r}({s})$.

(3) Because, as we have shown above, for all $v \in R$ we have $v \in {{\,\mathrm{CP}\,}}_{p}^{r}({s})$, by Definition 3, $v \in {{\,\mathrm{CP}\,}}_{p}^{r}({t})$. Therefore $\textsf {searchRoot}_p^r(t) = R$ by Lemma 5. $\square$

We continue with lemmas about the remaining helper functions. We show how they use the “getter-functions” $\textsf {L}()$ and $\textsf {X}()$ to check some crucial properties of the local state and approximation sets $\textsf {S}$, $\textsf {A}$.

##### Lemma 7

Let $\textsf {uniqueCandidate}_p^r(r-N, r-1) \ne -1$. Assume either of the following holds:

1. $$ \forall s \in [r-N, r-1], \forall q \in {{\,\mathrm{CP}\,}}_{p}^{r}({s}) :$$$$\ell _q^s = 0 \vee \textsf {x}_q^s = v$$, or
2. $$\exists s \in [r-N, r-1], \exists q \in {{\,\mathrm{CP}\,}}_{p}^{r}({s}) :$$$$\ell _q^s > 0 \wedge \textsf {x}_q^s = v$$,

then $$\textsf {uniqueCandidate}_p^r(r-N, r-1) = v$$.

##### Proof

Since $$\textsf {uniqueCandidate}_p^r(r-N, r-1) \ne -1$$, as stated in Line 24 of Algorithm 1, $$\exists k \in {\mathbb {N}} :$$$$\forall u \in \textsf {P}, \forall i \in [r-N, r-1] :$$$$\textsf {L}(u, i) > 0 \Rightarrow \textsf {X}(u, i) = k$$ and $$\exists q \in \textsf {P}, \exists j \in [r-N, r-1] :$$$$\textsf {L}(q, j) > 0$$. That is, by time *r*, *p* was influenced by a process *q* that was locked, i.e., had $$\ell _q > 0$$, in the last *N* rounds and in fact all locked processes that managed to influence *p* in the last *N* rounds were locked on to the same round *k*.

(1) By Lemmas 3 and 4 , $$\forall q \in \textsf {P}_p^r$$, $$\forall s \in [r-N, r-1] :$$$$\textsf {X}(q,s) = v$$ or $$\textsf {L}(q,s) \in \left\{ -1,0 \right\} $$. Thus, all processes that are locked, are locked on *v* and hence $$k=v$$. It follows that $$\textsf {uniqueCandidate}_p^r(r-N, r-1) = v$$.

(2) By Lemmas 3 and 4 , $\exists s \in [r-N, r-1], \exists q \in \textsf {P}_p^r$ with $\textsf {L}_p^r(q, s) > 0$ and $\textsf {X}_p^r(q, s) = v$. Hence $k=v$ and $\textsf {uniqueCandidate}_p^r(r-N, r-1) = v$. $\square$

##### Lemma 8

If $$\exists s \in [r-N, r-1], \exists q \in {{\,\mathrm{CP}\,}}_{p}^{r}({s})$$ with $$\ell _q^s > 0$$, and $$\forall s \in [r-N, r-1], \forall q \in {{\,\mathrm{CP}\,}}_{p}^{r}({s}) :$$$$\textsf {x}_q^s = v$$, then $$\textsf {uniqueCandidate}_p^r(r-N, r-1) = v$$.

##### Proof

By Lemmas 3 and 4 , $\exists q \in \textsf {P}_p^r$ with $\textsf {L}_p^r(q, s) > 0$ and $s \in [r-N, r-1]$, and $\forall q \in \textsf {P}_p^r$ we have $\textsf {L}_p^r(q, s) > 0 \Rightarrow \textsf {X}_p^r(q, s) = v$ for every $s \in [r-N, r-1]$. As stated in Line 24 of Algorithm 1, we therefore have $\textsf {uniqueCandidate}_p^r(r-N, r-1) = v$. $\square$

##### Lemma 9

Let $v = \textsf {x}_p^{r}$. The following all hold for all processes *p*:

1. If $q \in {{\,\mathrm{CP}\,}}_{p}^{r}({s})$ for a $s \in [r-N, r-1]$ and $\textsf {x}_q^s \ne v$ then $\textsf {latestRefutation}_p^r(r-N, r-1) \geqslant s$.
2. If $\textsf {x}_q^t = v$ and $\ell _q^t > 0$ for all $q \in {\varPi }_\sigma$ and all $t \in [s,r]$ then $\textsf {latestRefutation}_p^r(r-N, r-1) < s$.
3. $$\textsf {latestRefutation}_p^r(r-N, r-1) < r$$.

##### Proof

Let $\textsf {T}_p^r$ denote *p*’s value of $\textsf {T}$ during a call of $\textsf {latestRefutation}_p^r(r-N, r-1)$ after computing Line 20.

1. By Lemma 4, $\textsf {X}_p^r(q, s) \notin \left\{ -1, v \right\}$. Since $s \in [r-N, r-1]$, Line 20 of Algorithm 1 ensures that $s \in \textsf {T}_p^r$. Hence $\max (\textsf {T}_p^r)\geqslant s$ is returned in Line 21.
2. Suppose, $\textsf {latestRefutation}_p^r(r-N, r-1) = t \geqslant s$. By Line 20 of Algorithm 1, $t \in \textsf {T}_p^r$ with $\textsf {L}(q,t) = 0$ or $\textsf {X}(q,t) \notin \left\{ -1, v \right\}$ for a $q \in \textsf {P}_p^r$. According to Lemma 4, hence $\textsf {x}_q^t \ne v$ or $\ell _q^t = 0$, which contradicts our assumption.
3. Follows because $\textsf {T}_p^r \subseteq [r-N,r-1]$ by Line 20 of Algorithm 1. $\square$

##### Lemma 10

With $v = \textsf {x}_p^r$, $\textsf {allGood}_p^r(r-N(D+2N), r-1)$ is false if and only if $\exists s \in [r-N(D+2N),r-1]$ s.t. $q \in {{\,\mathrm{CP}\,}}_{p}^{r}({s})$ with $\ell _{q}^{s} = 0$ or $\textsf {x}_{q}^{s} \ne v$.

##### Proof

“If direction”: By Lemma 4, $\textsf {L}_p^r(q,s) = 0$ or $\textsf {X}_p^r(q,s) \notin \left\{ -1, v \right\}$ and, by Lemma 3, $q \in \textsf {P}_p^r$. Hence, the expression in Line 29 of Algorithm 1 is false.

“Only if direction”: If $\not \exists s \in [r-N(D+2N),r-1]$ s.t. $q \in {{\,\mathrm{CP}\,}}_{p}^{r}({s})$ with $\ell _{q}^{s} = 0$ or $\textsf {x}_{q}^{s} \ne v$, by Lemma 4 for all $s \in [r-N(D+2N),r-1]$, we have $\textsf {L}^r_p(q, s) \ne 0$ and $\textsf {X}^r_p(q, s) \in \left\{ -1, v \right\}$. Hence, Line 29 of Algorithm 1 evaluates to true. $\square$

#### Algorithm 2: Main consensus algorithm

We are now ready to formally prove that Algorithm 2 solves consensus. Throughout the remaining proofs, unless stated otherwise, all line numbers are w.r.t. Algorithm 2. We start with a formal argument for why an assignment $\textsf {x}_p \leftarrow v$ of process *p* in round *r* via Line 10 has the desired outcome of setting $\textsf {x}_p$ to the maximum proposal $x_q^{r-D}$ over all processes *q* that were member of the root component of ${\mathcal {G}}^{r-D}$.

##### Lemma 11

If *p* enters Line 10 in round *r* then $\textsf {x}_p^r = \max \{ \textsf {x}_q^{r-D} \mid q \in {{\,\mathrm{Root}\,}}({\mathcal {G}}^{r-D})\}$.

##### Proof

Since the guard of Line 9 has been passed, we have $\textsf {R}^r_p = \textsf {searchRoot}_p^r(r-D) \ne \emptyset$. Part (2) of Lemma 6 asserts that $\textsf {R}^r_p \subseteq {{\,\mathrm{CP}\,}}_{p}^{r}({r-D})$. By Lemma 4, for all $q \in \textsf {R}^r_p$, we hence have $\textsf {X}_p^r(q, r-D) = \textsf {x}_q^{r-D}$. Thus, after executing the assignment of Line 10, we have $\textsf {x}_p^r = \max \{ \textsf {X}_p^r(q, r-D) \mid q \in \textsf {R}^r_p \}$. This, combined with the observation that $\textsf {R}^r_p = {{\,\mathrm{Root}\,}}({\mathcal {G}}^{r-D})$ by item (1) of Lemma 6, asserts that $\textsf {x}_p^r = \max \{ \textsf {x}_q^{r-D} \mid q \in {{\,\mathrm{Root}\,}}({\mathcal {G}}^{r-D})\}$ as $x_p$ is not modified after this point. $\square$

For our analysis, we introduce the useful term *v-locked root component* to denote a round-*r* root component where all members *q* are *v-locked* for the same *v*, i.e., have the proposal $\textsf {x}^r_q = v$ and are locked, i.e., $\ell _q^r > 0$. In a *v-locked sequence*, every graph has a (possibly different) *v*-locked root component for the same value *v*.

##### Definition 8

$${{\,\mathrm{Root}\,}}({\mathcal {G}}^r)$$ is a *v*-locked root component if $$\forall p \in {{\,\mathrm{Root}\,}}({\mathcal {G}}^r) :$$$$\ell _p^r > 0$$ and $$\textsf {x}_p^r = v$$. A sequence $$({\mathcal {G}}^r)_{r \in I}$$ is *v*-locked if for all $$r \in I$$, $${{\,\mathrm{Root}\,}}({\mathcal {G}}^r)$$ is a *v*-locked root component.

The following technical lemma is key for the proof of the agreement property of consensus. The crucial part (3) assures that if a sequence of at least $D+2N$ communication graphs occurs in which all root components happen to be *v*-locked by our algorithm, then every process *p*’s proposal value is $x_p^r=v$ at every time *r* following this sequence.

##### Lemma 12

Let $\rho =({\mathcal {G}}^{r})_{r = a}^{b}$ be a *v*-locked sequence with $|\rho | = 2N+D$ and $\rho \subset \sigma \in \lozenge {{\textsf {STABLE}}}_{\leqslant N,D}(D+1)$. Then, all of the following hold for all processes $p \in {\varPi }_\rho$:

1. $$\forall r \in [a+N+D, b] :$$$$\textsf {x}^r_p = v \vee \ell _p^r = 0$$.
2. $$\forall r \geqslant b :\textsf {x}^r_p = v$$.

##### Proof

We observe from the code of Algorithm 2 that $\textsf {x}_p^r$ is only written to in Line 10 and Line 16.

(1) Pick an arbitrary $p \in {\varPi }_\rho$, $r \in [a+N+D, b]$. If $p \in {{\,\mathrm{Root}\,}}({\mathcal {G}}^r)$ the claim is immediate because ${{\,\mathrm{Root}\,}}({\mathcal {G}}^r)$ is *v*-locked by assumption. Otherwise, if *p* enters Line 10 or Line 16 at time *r*, we have $\textsf {x}_p^r = v$. In case of the former, this follows from Lemma 11 and because ${{\,\mathrm{Root}\,}}({\mathcal {G}}^{r-D})$ is *v*-locked by assumption. In case of the latter, because $\rho$ is *v*-locked, by Theorem 1, $\exists s \in [r-N, r-1], \exists q \in {{\,\mathrm{CP}\,}}_{p}^{s}({r}) :\ell _q^s >0 \wedge x_q^s = v$, and hence the claim holds due to (2) of Lemma 7. Hence, assume that none of these lines is entered and that $\textsf {x}_p^{r-1} \ne v$ and $\ell = \ell _p^{r-1} > 0$, as the claim follows immediately otherwise.

We find that then $\ell < a+D$, as an inductive argument shows that $\ell = 0 \vee \textsf {x}_p^{r-1} = v$ if not: If $\ell \geqslant a+D$, by Lemma 11 and because $\rho$ is *v*-locked, $\textsf {x}_p^\ell = v$. Consider time $s \in [\ell + 1, r-1]$ and assume the hypothesis $\ell _p^{s-1} = 0 \vee \textsf {x}_p^{s-1} = v$. If *p* enters Line 10, again by Lemma 11 and because $\rho$ is *v*-locked, $\textsf {x}_p^s = v$. If *p* enters Line 16 and $\textsf {x}_p^s = v' \ne v$, we have $\ell _p^{s-1} = \ell = 0$ because, if $\ell _p^{s-1} > 0$, then by hypothesis $\textsf {x}_p^{s-1} = v$ and hence $v' = v$ by item (2) of Lemma 7 and because $p \in {{\,\mathrm{CP}\,}}_{p}^{s-1}({s})$.

As Line 10 was not entered and clearly $r > N$, *p* passes the guard of Line 12. Since $\rho$ is *v*-locked, by Theorem 1, if $p \notin {{\,\mathrm{Root}\,}}({\mathcal {G}}^r)$, there is a $q \in {{\,\mathrm{CP}\,}}_{p}^{r}({s})$ and a $s \in [r-N, r-1] \subseteq [a+D, b]$ with $\ell _q^s > 0$ and $\textsf {x}_q^s = v$. But according to Lemma 9, we then have $\textsf {latestRefutation}_p^r(r-N,r-1) \geqslant s > \ell _p^{r-1}$ and thus Line 14 is executed, setting $\ell _p^r \leftarrow 0$.

(2) We use induction on $$r\geqslant b$$. For $$r=b$$, assume $$p \notin {{\,\mathrm{Root}\,}}({\mathcal {G}}^b)$$ as otherwise the claim follows because $$\rho $$ is *v*-locked. If *p* enters Line 10 in round *b*, $$x_p^b = v$$ because $$\rho $$ is *v*-locked. Otherwise, by Theorem 1, as $$p \notin {{\,\mathrm{Root}\,}}({\mathcal {G}}^b)$$, there is a $$s \in [r-N, r-1]$$ and a $$q \in {{\,\mathrm{CP}\,}}_{p}^{r}({s})$$ with $$\textsf {x}_q^s = v$$ and $$\ell _q^s > 0$$. By (1), $$\forall s \in [r-N, r-1],$$$$\not \exists q \in {{\,\mathrm{CP}\,}}_{p}^{r}({s}) :$$$$\textsf {x}_q^s \ne v$$ and $$\ell _q^s > 0$$. Hence, Line 16 is executed and $$\textsf {x}_p^b = v$$ by Lemma 8.

For $r \geqslant b$, by the induction hypothesis, it suffices to show that all modifications of $\textsf {x}_p$ at process *p* during round *r* do not invalidate the claim. If *p* enters Line 10 in round *r*, $\textsf {x}_p \leftarrow v$ is assigned according to Lemma 11 and because either $R = {{\,\mathrm{Root}\,}}({\mathcal {G}}^{r-D})$ is *v*-locked by assumption (for $r-D\geqslant b$), or else $q \in R \Rightarrow \textsf {x}_q^{r-D} = v$ by the induction hypothesis. If *p* passes the guard of Line 16, because of (1) and because of the induction hypothesis, we have $\textsf {x}^r_p = v$ due to item (1) of Lemma 7. $\square$

With these preparations, we can now prove agreement, validity and termination of our consensus algorithm.

##### Lemma 13

Algorithm 2 ensures agreement under each sequence $\sigma \in \lozenge {{\textsf {STABLE}}}_{\leqslant N,D}(D+1)$.

##### Proof

We show that if a process *p* decides *v* in round *r*, all future decisions are *v* as well. A decision $y_p^r \leftarrow v$ by *p* in round *r* can only occur if *p* executed Line 18, thus *p* was undecided, $r> N(D+2N)$, and $\textsf {allGood}_p^r(r-N(D+2N), r-1)$ is true. We show that this implies that there is a *v*-locked sequence $\sigma \subseteq ({\mathcal {G}}^{r})_{r = r-N(D+2N)}^{r-1} = \sigma '$ with $|\sigma |>2N+D$. This shows the claim as a decision by *q* in a round $s \geqslant r$ is based on $\textsf {x}_q^s$ and $\textsf {x}_q^s = v$ by item (2) of Lemma 12.

Suppose $\sigma '$ does not contain such a sequence $\sigma$. By the pigeonhole principle, there must be a set of rounds $S = \left\{ r_1, \ldots , r_N \right\} \subseteq [r-N(D+2N), r-1]$ such that for each $r_i \in S$, a $q_i \in {{\,\mathrm{Root}\,}}({\mathcal {G}}^{r_i})$ has $\ell _{q_i}^{r_i} = 0 \vee \textsf {x}_{q_i}^{r_i} \ne v$. Setting $f(i) = q_i$ and $G = \left\{ {\mathcal {G}}^{r_1}, \ldots , {\mathcal {G}}^{r_n} \right\}$ allows us to apply Theorem 1. There are two cases:

If $p = q_n$, $\textsf {x}_p^{r_n} \ne v \vee \ell _p^{r_n} = 0$. But then either the check $\ell _p^r > 0$ fails (if $r=r_n$) or $\textsf {allGood}_p^r(r-N(D+2N), r-1)$ is false by Lemma 10 and because $p \in {{\,\mathrm{CP}\,}}_{p}^{r}({r_n})$ (if $r > r_n$).

If $p \ne q_n$, by Theorem 1, $\exists i \in [1,n] :q_i \in {{\,\mathrm{CP}\,}}_{p}^{r}({r_i})$ with $\ell _{q_i}^{r_i} = 0 \vee \textsf {x}_{q_i}^{r_i} \ne v$. Thus, again by Lemma 10, $\textsf {allGood}_p^r(r-N(D+2N), r-1)$ is false. $\square$

##### Lemma 14

Algorithm 2 ensures validity under all sequences $\sigma$ of $\lozenge {{\textsf {STABLE}}}_{\leqslant N,D}(D+1)$.

##### Proof

Validity follows from an induction on the time *r*, as all processes *p* decide on the value of $\textsf {x}_p^r$.

Initially, $\forall p \in {\varPi }_\sigma :\textsf {x}_p^0 = x_p$ where $x_p$ is the input value of process *p* by Line 1.

For all times $$r > 0$$ let the hypothesis be $$\forall p \in {\varPi }_\sigma , \forall s \in [0, r-1] :$$$$\textsf {x}_p^s = x_q$$ for an input value $$x_q$$. Assume *p* assigns $$\textsf {x}_p \leftarrow v$$ in round *r*. As this assignment is via Line 10 or Line 16, $$v = \textsf {X}_p^r(q, s) \ne -1$$ for a round $$s < r$$ and a process *q*. By Lemma 4, $$v = \textsf {x}_q^s$$, which is the input $$x_{q'}$$ of a process $$q'$$ by the induction hypothesis. $$\square $$

##### Lemma 15

Algorithm 2 terminates under all sequences $\sigma$ of $\lozenge {{\textsf {STABLE}}}_{\leqslant N,D}(D+1)$.

##### Proof

Since $\sigma \in \lozenge {{\textsf {STABLE}}}_{\leqslant N,D}(D+1)$, there is an earliest subsequence $\sigma ' = ({\mathcal {G}}^{r})_{r = a}^{b} \subset \sigma$ with $|\sigma '| = D+1$ that has a common root *R* and hence, for $a > 1$, $R' = {{\,\mathrm{Root}\,}}({\mathcal {G}}^{a-1}) \ne R$; for $a=1$, we just set $R'=\emptyset$. Let $v = \max \lbrace \textsf {x}_q^a \mid q \in R \rbrace$. We show by induction that for all processes *p* and for all times $r \geqslant b$, $\ell _p^r \geqslant b$, and $\textsf {x}_p^r = v$. This shows the claim as, at latest in round $s = b+N(D+2N)$, $\textsf {allGood}^s_p(b, s-1)$ is true by Lemma 10 at every undecided process *p*, leading to *p* deciding via Line 18.

In round *b*, for all processes *p*, $\textsf {searchRoot}_p^b(b-D) = R \ne \emptyset$ by (2) of Lemma 5. Furthermore, $\textsf {searchRoot}_p^b(b-D-1) = \emptyset$ if $R'=\emptyset$ and $\textsf {searchRoot}_p^b(b-D-1) = R' \ne R$ by Lemma 6. Hence *p* passes Line 9 and enters Line 10 and Line 11. Thus $\ell ^b_p = b$, and, by Lemma 11, $\textsf {x}^b_p = v$.

Pick a round $r > b$. Using the induction hypothesis, it suffices to show that a modification of $\ell _p$ resp. $\textsf {x}_p$ in round *r* does not invalidate our claim.

If either is modified by Line 10 or Line 11, since by the hypothesis $\ell _p^{r-1} > 0$, we must have $\textsf {searchRoot}_p^r(r-D-1) \ne \textsf {searchRoot}_p^r(r-D)$. As $\textsf {searchRoot}_p^r(b-D) = R \ne \emptyset$ and $\sigma ' = ({\mathcal {G}}^{i})_{i = a}^{b}$ is $R$-rooted, by item (3) of Lemma 6, $r > b+D$. By the hypothesis, thus $\textsf {x}_q^{r-D} = v$ for all $q \in {{\,\mathrm{Root}\,}}({\mathcal {G}}^{r-D})$ and hence $\textsf {x}_p^r = v$ by Lemma 11.

If $\ell _p$ is set to 0 in Line 14, recall that the induction hypothesis guarantees $\textsf {x}_q^s = v$ and $\ell _q^s \geqslant b$ for all processes *q* and every time $s \in [b,r-1]$. Thus $\textsf {latestRefutation}(r-N, r-1) < b$ by Lemma 9 (2), and the check in Line 13 fails.

If $\textsf {x}_p$ is modified by Line 16, we have $\textsf {x}_p^r = v$ because of (2) of Lemma 7, as process *p* itself satisfies $p \in {{\,\mathrm{CP}\,}}_{p}^{r-1}({r})$ by Definition 3, and $\ell _p^{r-1} \geqslant b$ and $\textsf {x}_p^{r-1} = v$ by the hypothesis.

$$\square $$

The correctness of Algorithm 2 follows from Lemmas 13, 14, and 15:

##### Theorem 4

Algorithm 2 solves consensus under all $\sigma \in \lozenge {{\textsf {STABLE}}}_{\leqslant N,D}(D+1)$.

## Discussion and open questions

We provided tight upper and lower bounds for the solvability of consensus under message adversaries that guarantee a stable root component only eventually and only for a short period of time: We showed that consensus is solvable if and only if each graph has exactly one root component and, eventually, there is a period of at least $D+1$ consecutive rounds (with $D\leqslant n-1$ denoting the number of rounds required by root members for broadcasting) where the root component remains the same. We also provided a matching consensus algorithm, along with its correctness proof.

Regarding solutions to uniform consensus, where the number of processes is unknown, Theorem 2 showed that uniform consensus is impossible if the duration of the stability period is $<2D$. For a stability period with duration $>2D$, [24] presents a uniform consensus algorithm that works even under assumptions on the communication graphs that are more relaxed than to assume that all of them are rooted. This means that, even though almost the entire range of durations of the stability window w.r.t. the solvability of uniform consensus is now explored, the question of whether a stability duration of exactly 2*D* rounds is sufficient, remains open.

The aim of the paper was to answer the fundamental question of solvability of consensus in rooted dynamic networks with respect to the minimal duration of an eventually stabilizing root component. We answered this question by means of our impossibility results and Algorithm 2. While this shows that a stability period duration of $D+1$ rounds is a tight bound for consensus solvability in rooted dynamic networks, there is little focus on speed or efficiency.

One aspect that therefore might warrant further study, is the dependence of Algorithm 2 on the knowledge of the system size. As we know, counting the number of processes is hopeless under a message adversary in general (there can always be an arbitrarily long outgoing chain from a process), yet knowledge of *n* alone is insufficient to solve consensus, as we have seen in Theorem 3. Still, Algorithm 2 shows that exact knowledge of *n* is not necessary under the message adversary $\lozenge {{\textsf {STABLE}}}_{\leqslant N,D}$, and an estimate $N \geqslant n$ suffices. It remains an open question, how far this relaxation can be taken and whether agreement on *N* is in fact necessary or whether there is an algorithm that can even cope with each process *p* holding a different estimate $N_p \geqslant n$.

Another topic is related to the termination time of Algorithm 2. From the proof of Lemma 15, we can see that the algorithm terminates $O(N^2)$ rounds after the beginning of the stability period, yet it remains unclear, whether a faster termination time is achievable. We conjecture that information propagation after the first $D+1$ rounds of stability is crucial to all solution algorithms and therefore an *o*(*N*) solution would be very surprising. Nevertheless, investigating the existence of an efficient *O*(*N*) algorithm seems like a promising avenue for future work.

## Footnotes

1 Nevertheless, a crash of a process *p* in a round could easily be modelled by *p* sending no messages in a later round.

2 It assumes that all processes simultaneously broadcast a message at the beginning of a round, receive the messages from each other, and then simultaneously make a state transition at the end of the round, thereby proceeding to the next round. All these activities will be assumed to take place within the round, which results in well-defined and stable system states between rounds.

3 Whereas this may seem a somewhat unrealistic (though inevitable) restriction at first sight, it must be noted that *D* only needs to be guaranteed throughout the stability interval.

4 We sometimes write $p \in {\mathcal {G}}^r$ instead of $p \in {\varPi }$ to stress that *p* is a vertex of ${\mathcal {G}}^r$, and sloppily write $({p}, {q}) \in {\mathcal {G}}^r$ instead of $({p}, {q}) \in E^r$.

5 As we will see in Sect. 5 de facto the processes require only knowledge of some key parameters.

6 As we will see in the next section, consensus is possible under $\lozenge {{\textsf {STABLE}}}_{n,D}(D+1)$ even though in some cases (e.g. if $D=n-1$ and all communication graphs are chains) up to $(n-1)^2$ messages may be lost in each round!
